# Relationship of Serum IL-12 to Inflammation, Hematoma Volume, and Prognosis in Patients With Intracerebral Hemorrhage

**DOI:** 10.1155/2022/8688413

**Published:** 2022-10-18

**Authors:** Yazhao Zhang, Yanan Tian, Jianhui Wei, Yi Xiang

**Affiliations:** ^1^Department of Neurosurgery, Hengshui People's Hospital, Hengshui 053800, China; ^2^Department of Neurology, Hengshui People's Hospital, Hengshui 053800, China

## Abstract

**Objective:**

Inflammatory cascades and hematomas after intracerebral hemorrhage (ICH) cause brain tissue and neuronal damage. Interleukin-12 (IL-12) promotes brain inflammation, and regulates coagulation mediated by red blood cells and platelets. This study was designed to investigate the relationship of serum IL-12 to inflammation, hematoma volume, and prognosis in ICH patients.

**Methods:**

We recruited patients with ICH within 12 hours of symptom onset (*n* = 209) and measured their serum IL-12 levels. Patients with an increased National Institute of Health stroke scale (NIHSS) score ≥4 were defined as early neurological deterioration, and modified rankin scale (mRS) score >2 at 3 months after intracerebral hemorrhage was defined as poor prognosis.

**Results:**

Levels of serum IL-12 was positively correlated with the admission of NIHSS scores (*r* = 0.535, *P* < 0.001), hematoma volume (*r* = 0.608, *P* < 0.001), serum CRP levels (*r* = 0.561, *P* < 0.001), and serum TNF-*α* levels (*r* = 0.533, *P* < 0.001) in 209 cases ICH patients. Levels of IL-12 in ICH patients with early neurological deterioration (median: 82.9 versus 65.8, *P* < 0.001) or with poor prognosis (median: 79.0 versus 65.3, *P* < 0.001) were all significantly higher than those in other ICH patients. In addition, serum IL-12 levels could be used to differentiate ICH patients at risk for early neurological deterioration with an AUC of 0.788 (95% CI: 0.717–0.858) or ICH patients at risk for suffering from an unfavorable outcome with an AUC of 0.787 (95% CI: 0.722–0.851).

**Conclusion:**

Elevated admission serum IL-12 levels are closely related to the inflammation, hematoma volume, and prognosis in ICH patients. Substantializing serum IL-12 levels is a prognostic biomarker for ICH.

## 1. Introduction

Intracerebral hemorrhage (ICH) is a nontraumatic hemorrhage caused by rupture of blood vessels in the brain parenchyma, accounting for about 20% to 30% of cerebrovascular diseases, and ICH is characterized by high morbidity, high mortality, and a high disability rate [1, 2]. Clinical data shows that about 88% of surviving ICH patients are left with significant disability, which brings a serious burden to society and families [3]. After an intracerebral hemorrhage, the hematoma will compress normal brain tissue and cause primary ischemic brain injury. At the same time, primary brain injury caused by hematoma mass disrupts the blood-brain barrier, and hematoma metabolites or lysed erythrocyte products can cause secondary brain injury by causing oxidative stress, inflammation, cytotoxicity, and excitotoxicity [4, 5]. Inflammatory response plays an important role in the whole period of ICH-induced brain injury, including primary brain injury and secondary brain injury [6, 7]. Inflammatory cells, including leukocytes, glial cells/macrophages, astrocytes, and their released various cytokines, chemokines, enzymes, and immunologically active small-molecule polypeptides secreted, are involved in the inflammatory cascade post-intracerebral hemorrhage [8, 9]. They all play an important role in the inflammatory cascade and are also considered to be important biomarkers for predicting the prognosis of patients after intracerebral hemorrhage [8, 9].

Interleukin-12 (IL-12) is the earliest discovered member of the IL-12 cytokine family, mainly derived from dendritic cells, macrophages, and *B* lymphocytes [10, 11]. It is a sexual factor that connects the early nonspecific innate immunity and the late antigen-specific adaptive immunity and participates in the immune regulation of infection, tumor, and autoimmune diseases [12, 13]. In conclusion, IL-12 is a pro-inflammatory factor after inflammation occurs [14, 15]. Previous studies have shown that the expression of IL-12 is elevated around the hematoma in animal models of cerebral hemorrhage, and the use of IL-12 receptor monoclonal antibody can alleviate brain injury after intracerebral hemorrhage. Its mechanism is thought to be involved in regulating macrophage activation [16]. However, no study has investigated serum IL-12 levels in patients with ICH, nor has there been a study investigating the relationship between serum IL-12 levels and circulating inflammation, hematoma volume, early neurological deterioration, and 3-month prognosis in patients with ICH. In the present study, we recruited patients with ICH, quantified serum IL-12 levels, and analyzed the association of serum IL-12 levels with circulating inflammation, hematoma volume, early neurological deterioration, and 3-month prognosis.

## 2. Materials and Methods

### 2.1. Study Population

From January 2016 to December 2019, there are 209 patients with ICH being prospectively recruited into the present study at Hengshui People's Hospital. These ICH patients must meet the following standards. Inclusion criteria: (1) age ≥18 years old; (2) underwent brain CT scans immediately at admission and being diagnosed as ICH; (3) recruited within 12 hours of symptom onset of intracerebral hemorrhage; (4) underwent laboratory examination at admission. Exclusion criteria: (1) ICH induced by trauma; (2) ICH caused by brain tumors, vascular malformations, moyamoya disease, and brain aneurysms; (3) History of the following medications and treatments within 6 months of being recruited: antiplatelet, anticoagulant therapy, anti-inflammatory drugs, steroids, or immunosuppressants; (4) history of surgery within one month; (5) malignant tumors, systemic diseases, chronic infectious diseases, autoimmune diseases, pregnant women, lactating women, coagulation disorders, and menstruating women. At the same time, we recruited 100 healthy volunteers as baseline controls for ICH patients. This study complies with the principles of the Declaration of Helsinki and was reviewed and approved by the Ethics Committee of Hengshui People's Hospital (No. of ethical approval: 2019-02-011).

### 2.2. Data Collection

We collected the clinical data of patients at admission, including age, gender, admission time, blood collection, initial systolic blood pressure (SBP), initial diastolic blood pressure (DBP), medication history, medical history, hospitalization history, hematoma volume and hematoma location calculated from CT scan, laboratory test data, comorbidities (hypertension), and living habits (smoking). The severity of stroke was assessed by the National Institute of Health stroke scale (NIHSS) score in patients with ICH at admission, and the NIHSS score was performed on ICH patients every 3 hours thereafter. An increase in the NIHSS score ≥4 was defined as early neurological deterioration [17, 18]. All ICH patients were followed for 3 months after intracerebral hemorrhage or death. At 3 months after intracerebral hemorrhage, the modified rankin scale (mRS) was used to evaluate the outcome after the hemorrhage, and a mRS score >2 at 3 months after intracerebral hemorrhage was defined as poor prognosis [17].

### 2.3. Serum IL-12 Detection

Peripheral blood samples were drawn from ICH patients at admission, followed by centrifugation (1000 × *g*, room temperature, 10 minutes) to collect serum. And then serum was stored at −80°C for subsequent testing. Serum IL-12 levels were quantified in three duplicates using a human IL-12 enzyme-linked immunosorbent assay kit (QS40063, Beijing Qisong Biotechnology Co., Ltd., China). Determinations were completed in batches by the same technician blinded to clinical data.

### 2.4. Statistical Analysis

Data in the present study were analyzed by SPSS 19.0 software (SPSS Inc., Chicago, USA). Qualitative data are presented as counts (%), and *P*values are calculated using the chi-square or Fisher's exact test as appropriate. Kolmogorov–Smirnov test was used to check whether quantitative data conformed to a normal distribution, data that conformed to a normal distribution were presented as (mean ± standard deviation), and unpaired Student's *t*-test was used to compare differences and calculate *P*values. Quantitative data that did not conform to a normal distribution are presented as the median (interquartile range), and Mann–Whitney *U*-test was used to compare differences and calculate *P*values. Spearman's correlation coefficient was used to analyze the association of serum IL-12 levels with other clinical features. Receiver operating characteristic (ROC) curves were constructed and the area under the curve (AUC) was calculated to assess the performance of serum IL-12 levels in distinguishing between ICH patients with and without early neurological deterioration or in distinguishing between ICH patients with and without poor outcom at 3 months after intracerebral hemorrhage.

## 3. Results

### 3.1. Demographics and Characteristics of Study Subjects and Serum IL-12 Levels

We evaluated 209 patients with ICH (Figure ) and selected 100 healthy volunteers as baseline controls. The 209 ICH patients included 121 males and 88 females aged (66.7 ± 18.7) years. Hypertension, current smoking, and atrial fibrillation were found in 135 (64.6%) cases, 67 (32.1%) cases and 46 (22.0%) cases, respectively. Herein, the admission time of ICH patients were from 1 to 22 hours (median: 5.8 h, interquartile range (IQR): 7.0 h), the time of blood collection were acquired from 4.5 h to 23.5 h (median: 6.9 h, IQR: 10.2 h), and the initial systolic blood pressure and diastolic blood pressure were (160.3 ± 27.3) mmHg and (91.5 ± 18.4) mmHg, respectively. The site of bleeding in patients with ICH was lobar hemorrhage, infratentorial hemorrhage, and intraventricular hemorrhage being found in 45 (21.5%) cases, 37 (17.7%) cases and 49 (23.4%) cases, respectively. The admission NIHSS scores were from 1 to 19 (median: 10, IQR: 10), the hematoma volume was from 3.2 mL to 66.7 mL (median: 16.9 mL, IQR: 14.8 mL). The value of white blood cell count, serum glucose concentration in ICH patients were (9.6 ± 3.1) × 10^9^/L and (7.4 ± 1.3) mmol/L. The serum levels of CRP, TNF-*α* and IL-12 were from 6.5 mg/L to 37.6 mg/L (median: 22.3 mg/L, IQR: 10.0 mg/L), 19.7 pg/mL to 90.8 pg/mL (median: 63.2 pg/mL, IQR: 23.2 pg/mL) and 29.6 pg/mL to 121.4 pg/mL (median: 71.5 pg/mL, IQR: 28.5 pg/mL). There was no significant difference in age and gender between ICH patients and healthy controls, but the values of white blood cell count and serum levels of glucose, CRP, TNF-*α* and IL-12 in ICH patients were all significantly higher than those in healthy controls (Table 1).

### 3.2. Correlation of Serum IL-12 Levels with Clinical Features, Inflammation, and Hematoma Volume

**Figure 1:**
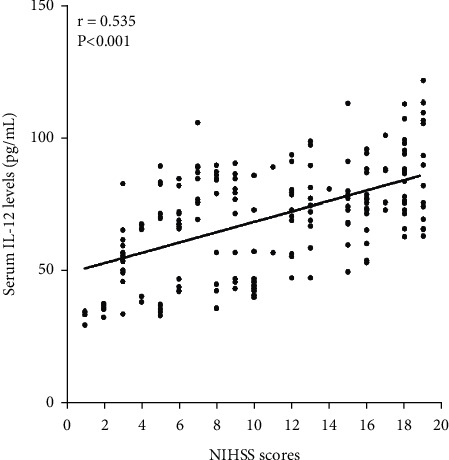
Scatter plot showing the correlation of serum IL-12 levels with NIHSS scores using Spearman's correlation coefficient.

**Figure 2:**
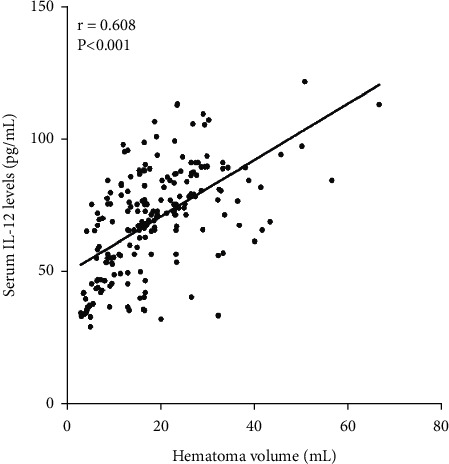
Scatter plot showing the correlation of serum IL-12 levels with hematoma volume using Spearman's correlation coefficient.

**Figure 3:**
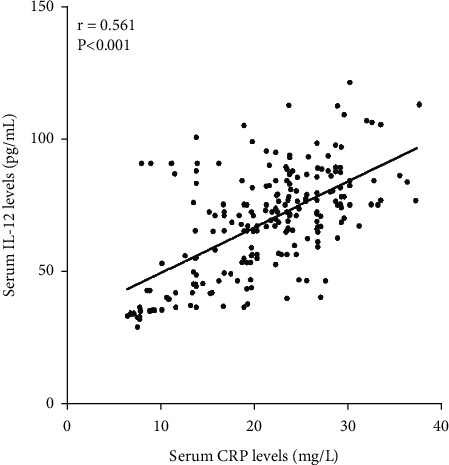
Scatter plot showing the correlation of serum IL-12 levels with serum CRP levels using Spearman's correlation coefficient.

**Figure 4:**
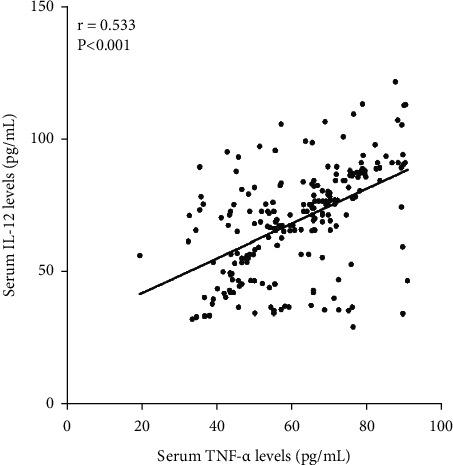
Scatter plot showing the correlation of serum IL-12 levels with serum TNF-*α* levels using Spearman's correlation coefficient.

We analyzed the correlation of serum IL-12 levels with other clinical features using Spearman correlation coefficient in 209 cases ICH patients, and the results showed that serum IL-12 level was closely related to admission NIHSS scores (*r* = 0.535, *P* < 0.001, Figure 1), hematoma volume (*r* = 0.608, *P* < 0.001, Figure 2), serum CRP levels (*r* = 0.561, *P* < 0.001, Figure 3), and serum TNF-*α* levels (*r* = 0.533, *P* < 0.001, Figure 4) in 209 cases ICH patients (Table 2).

### 3.3. Predictive Analysis of Serum IL-12 on Early Neurological Deterioration

**Figure 5:**
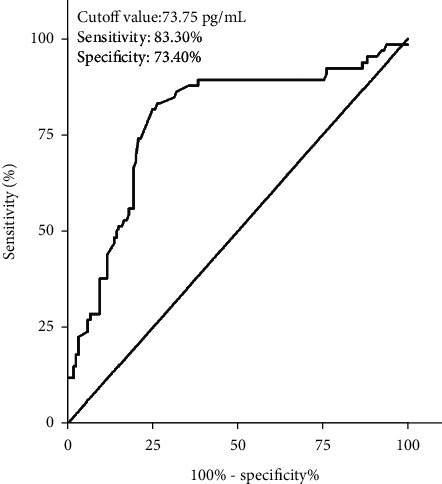
ROC curve analysis regarding predictive value of serum IL-12 levels for early neurologic deterioration after intracerebral hemorrhage.

Among 209 patients with ICH, 66 (31.6%) patients with ICH had early neurological deterioration, and then we compared the clinical characteristics of ICH patients with and without early neurological deterioration. The results showed that there was no significant difference in gender, admission time, blood collection time, initial SBP, initial DBP, hypertension, current smoking, atrial fibrillation, and hemorrhage site between ICH patients with and without early neurological deterioration, but age, the admission NIHSS score, hematoma volume, WBC, and the levels of serum CRP, TNF-*α*, and IL-12 in ICH patients with early neurological deterioration were significantly higher than those in ICH patients without early neurological deterioration (Table 3). Moreover, the results of ROC curve analysis showed that serum IL-12 levels could be used to differentiate patients at risk for early neurological deterioration with an AUC of 0.788 (95% CI: 0.717–0.858) (Figure 5). The serum IL-12 level of 73.75 pg/mL was used as the cutoff value to distinguish the early neurological deterioration of ICH patients, and the sensitivity of serum IL-12 in the diagnosis of early neurological deterioration in patients with ICH is 83.30% and the specificity is 73.40% (Figure 5).

### 3.4. Predictive Analysis of Serum IL-12 on 3-Month Poor Outcome after Hemorrhage

**Figure 6:**
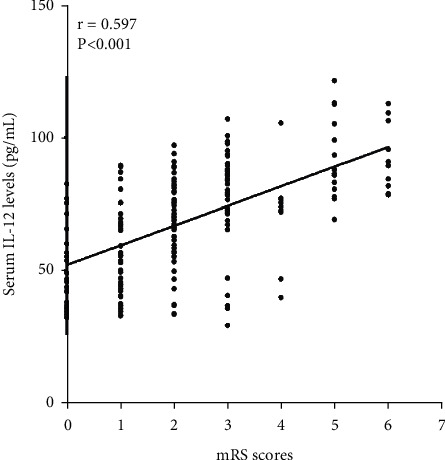
Scatter plot showing the correlation of serum IL-12 levels with mRS scores followed by intracerebral hemorrhage using spearman correlation coefficient.

**Figure 7:**
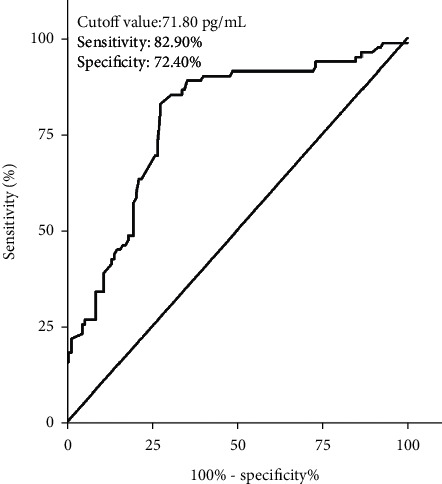
ROC curve analysis regarding predictive value of serum IL-12 levels for 3-month poor outcome after intracerebral hemorrhage.

3 months after intracerebral hemorrhage, we used the modified Rankin scale score (mRS) to assess the prognosis of ICH patient, and 23, 46, 58, 40, 17, 15, and 10 patients had mRS score 0, 1, 2, 3, 4, 5 and 6, respectively. And there was a positive correlation between the mRS score at 3 months after intracerebral hemorrhage and the serum levels of IL-12 (*r* = 0.597, *P* < 0.001, Figure 6). According to the agreement (ICH patients suffered from an unfavorable outcome when mRS score >2), 82 (39.2%) of the ICH patients' cases suffered from an unfavorable outcome. And then we compared the clinical characteristics of ICH patients with and without suffering from an unfavorable outcome. Results showed that there was no significant difference in gender, admission time, blood collection time, initial SBP, initial DBP, hypertension, current smoking, atrial fibrillation, and hemorrhage site between ICH patients with and without suffering from an unfavorable outcome, but age, the admission NIHSS score, hematoma volume, WBC, and the levels of serum CRP, TNF-*α*, and IL-12 in ICH patients with early neurological deterioration were significantly higher than that in ICH patients without early neurological deterioration (Table 4). In addition, the result of ROC curve analysis showed that serum IL-12 levels could be used to differentiate patients at risk for suffering from an unfavorable outcome with an AUC of 0.787 (95% CI: 0.722–0.851) (Figure 7). When the serum IL-12 level of 71.80 pg/mL was used as the cutoff value to distinguish the 3-month poor outcome after hemorrhage, and the sensitivity of serum IL-12 in the diagnosis of the 3-month poor outcome after hemorrhage in patients with ICH is 82.90% and the specificity is 72.40% (Figure 7).

## 4. Discussion

The IL-12 protein family is a class of heterodimeric cytokines, including IL-12, IL-23, IL-27, and IL-35, that exert many biological effects in the regulation of the immune system [12, 13]. IL-12 is a 70 kDa heterodimeric cytokine composed of covalently linked p40 and p35 subunits that has been shown to be a Th1-related proinflammatory cytokine [14, 15]. IL-12 is mainly derived from B lymphocytes, macrophages, and dendritic cells with antigen-presenting ability [14, 15]. Lots of previous studies have shown that IL-12 plays an important role in the occurrence and development of various inflammatory diseases, such as inflammatory bowel disease [19], psoriasis [20], chronic enteritis [21], allergic asthma [22], and so on. At the same time, IL-12 is also involved in the regulation of many inflammatory cascades in ischemic and hypoxic diseases [23, 24]. As a disease closely related to inflammation, we speculate that IL-12 may be involved in the inflammatory cascade after intracerebral hemorrhage. Consistently, IL-12 was found to be highly expressed in the brain tissue of animal models of cerebral hemorrhage and blocking the activity of IL-12 by antibodies could reduce inflammation and attenuate brain damage caused by cerebral hemorrhage [16].

In the present study, we not only found that IL-12 was elevated in the serum of ICH patients but also found that serum IL-12 levels were positively correlated with serum CRP and TNF-*α* levels in ICH patients, suggesting that serum IL-12 level was related to peripheral blood inflammation in ICH patients. Cytokines act as major players in information transduction between different cells, coordinating the balance of various pro- and anti-inflammatory cell populations, acting as growth factors or initiating differentiation and maturation [25, 26]. As an important immune regulator, IL-12 is a cytokine with multiple functions, and its main target cells are T lymphocytes and NK cells [27]. IL-12 exerts a wide range of biological activities by combining with its receptors, which can enhance the cytotoxic effect of cytotoxic *T* cells and other cells, induce the proliferation and activation of NK cells and *T* lymphocytes, promote the generation of Th1 cells, inhibit the generation of Th2, and stimulate the secretion of various cytokines, including TNF-*α* [28, 29]. In addition, the changes of serum IL-12 levels and CRP levels are synchronized in many clinical investigations [30, 31]. Therefore, serum IL-12 level may be related to the inflammatory response after intracerebral hemorrhage and participate in the regulation of inflammation-mediated brain tissue damage after intracerebral hemorrhage, but its specific role needs further research and verification.

The area of tissue lesions that forms edema around the hematoma after intracerebral hemorrhage is called the penumbra or hematoma penumbra, and neuronal apoptosis or necrosis caused by hypoxia or reperfusion in this area is a key factor affecting patients with ICH [32, 33]. The influence of hematoma volume and subsequent hematoma volume changes after intracerebral hemorrhage is the most important key on the area of penumbra and cell damage [32, 33]. The main symptoms of patients with cerebral hemorrhage are cerebral edema around the hematoma, abnormal metabolism around the hematoma, and hematoma in the brain tissue. The hematoma formed after intracerebral hemorrhage can not only cause space-occupying damage to the surrounding tissues but also the exuded blood components and their cleavage products can cause toxic damage to the surrounding tissues, especially the peripheral cerebral edema caused by the hematoma can further lead to secondary neurological deficits, which is very detrimental to the development of the disease [4, 5]. More and more evidence shows that blood coagulation, blood clot retraction and hematoma mass effect caused by the combined action of red blood cells, white blood cells, hemoglobin, iron ions and various cytokines are closely related to the formation of peri-hematoma brain edema [34, 35]. Interestingly, a recent study found that IL-12 can promote coagulation by promoting platelet activation and proliferation, promoting red blood cell agglutination and increasing fibrin content [36], which suggests IL-12 may be related to the formation and changes of hematoma after intracerebral hemorrhage, as well as the neurological deficit and prognosis of ICH patients caused by hematoma.

Fortunately, we found that serum IL-12 levels were positively correlated with intracerebral hemorrhage severity and hematoma volume. Subsequently, the results of univariate analysis of variables showed that serum IL-12 level was closely associated with early neurological deficits and 3-month prognosis after ICH. Furthermore, the results of ROC curve analysis showed serum IL-12 level could be used to differentiate early neurological deficits and 3-month prognosis in ICH patients. All in all, our data showed that serum IL-12 level was an inflammatory marker after intracerebral hemorrhage and might serve as a biomarker for predicting prognosis in patients with intracerebral hemorrhage. However, three important limitations of this study should be noted. First, patients with ICH have received different therapies, including surgical removal of the hematoma, mannitol, albumin, and antibiotics, which may have different effects on patient outcomes. Second, *P* < 0.05 was considered statistically significant in this study, but there were some significant differences in *P* values between 0.05 and 0.01 in this study. Therefore, the statistical significance may not be large enough. Finally, only 10 ICH patients out of 209 scored as 6 by the Rankin scale score scale at 3-month follow-up in the present study, which is lower than the study by Tu et al. [37], so the biomarker conclusion of IL-12 needs further clinical large sample verification.

## 5. Conclusion

This study is the first to quantify serum IL-12 levels in patients with cerebral hemorrhage and analyze their relationship with the severity of cerebral hemorrhage, peripheral blood inflammation, early neurological deficit, and 3-month prognosis in patients with cerebral hemorrhage. In the present study, elevated admission serum IL-12 levels are closely related to the inflammation (CRP and TNF-*α*), hematoma volume, and prognosis in ICH patients, substantializing serum IL-12 levels is an inflammation and prognostic biomarker for ICH.

## Figures and Tables

**Table 1:** Demographics and serum IL-12 levels in controls and patients.

Variables	Control (*n* = 100)	Patients (*n* = 209)	*P* value
Gender (male/female)	54/46	121/88	0.518
Age (years)	65.9 ± 21.3	66.7 ± 18.7	0.812
Admission time (hours)	—	5.8 (7.0)	—
Blood collection time (hours)	—	6.9 (10.2)	—
Initial SBP (mmHg)	—	160.3 ± 27.3	—
Initial DBP (mmHg)	—	91.5 ± 18.4	—
Hypertension (*n* (%))	—	135 (64.6)	—
Current smoking (*n* (%))	—	67 (32.1)	—
Atrial fibrillation (*n* (%))	—	46 (22.0)	—
Lobar hemorrhage (*n* (%))	—	45 (21.5)	—
Infratentorial hemorrhage (*n* (%))	—	37 (17.7)	—
Intraventricular hemorrhage (*n* (%))	—	49 (23.4)	—
Admission NIHSS	—	10 (10)	—
Hematoma volume (mL)	—	16.9 (14.8)	—
WBC (×10^9^/L)	8.9 ± 2.3	9.6 ± 3.1	0.039
Serum glucose (mmol/L)	7.1 ± 1.2	7.4 ± 1.3	0.043
Serum CRP (mg/L)	2.0 (0.8)	22.3 (10.0)	0.041
Serum TNF-*α* (pg/mL)	28.6 (26.3)	63.2 (23.2)	<0.001
Serum IL-12 (pg/mL)	20.4 (19.1)	71.5 (28.5)	<0.001

**Table 2:** Correlation between serum IL-12 levels and other variables using Spearman's correlation coefficient in ICH patients.

Variables	*r*	*P* value
Gende	0.036	0.812
Age	0.092	0.767
Admission time	−0.135	0.328
Blood collection time	−0.057	0.793
Initial SBP	0.158	0.304
Initial DBP	0.143	0.315
Hypertension	0.062	0.799
Current smoking	0.091	0.772
Atrial fibrillation	0.038	0.811
Lobar hemorrhage	−0.064	0.803
Infratentorial hemorrhage	0.163	0.296
Intraventricular hemorrhage	0.082	0.784
Admission NIHSS	0.535	<0.001
Hematoma volume	0.608	<0.001
WBC	0.301	0.052
Serum glucose	0.257	0.081
Serum CRP	0.561	<0.001
Serum TNF-*α*	0.533	<0.001

**Table 3:** Factors associated with early neurological deterioration in patients with ICH.

Variables	Early neurological deterioration		*P* value
	Yes (*n* = 66)	No (*n* = 143)	
Gender (male/female)	40/26	81/62	0.590
Age (years)	70.2 ± 15.4	65.1 ± 21.3	0.0035
Admission time (hours)	6.2 (9.5)	5.6 (8.9)	0.426
Blood collection time (hours)	7.2 (7.1)	6.8 (7.3)	0.194
Initial SBP (mmHg)	162.3 ± 23.5	159.4 ± 26.8	0.438
Initial DBP (mmHg)	93.8 ± 19.6	90.4 ± 23.2	0.208
Hypertension (*n* (%))	47 (71.2)	88 (61.5)	0.174
Current smoking (*n* (%))	22 (33.3)	45 (31.5)	0.788
Atrial fibrillation (*n* (%))	18 (27.3)	28 (19.6)	0.212
Lobar hemorrhage (*n* (%))	18 (27.3)	27 (18.9)	0.170
Infratentorial hemorrhage (*n* (%))	12 (18.2)	25 (17.5)	0.902
Intraventricular hemorrhage (*n* (%))	17 (25.8)	32 (22.4)	0.592
Admission NIHSS	16.0 (9.0)	8.0 (8.0)	<0.001
Hematoma volume (mL)	21.6 (12.3)	16.5 (14.3)	0.005
WBC (×10^9^/L)	10.8 ± 2.5	9.0 ± 2.6	0.032
Serum glucose (mmol/L)	8.2 ± 1.5	7.0 ± 1.1	0.019
Serum CRP (mg/L)	26.7 (5.68)	19.7 (9.8)	<0.001
Serum TNF-*α* (pg/mL)	70.0 (14.8)	56.2 (21.4)	<0.001
Serum IL-12 (pg/mL)	82.9 (14.9)	65.8 (26.3)	<0.001

**Table 4:** Factors associated with 3-month poor outcome in patients with ICH.

Variables	Early neurological deterioration		*P* value
	mRS (3–6) (*n* = 82)	mRS (0–2) (*n* = 127)	
Gender (male/female)	50/32	71/56	0.469
Age (years)	69.3 ± 16.8	66.1 ± 15.4	<0.001
Admission time (hours)	6.0 (8.3)	5.8 (7.2)	0.389
Blood collection time (hours)	7.0 (7.3)	6.9 (7.2)	0.441
Initial SBP (mmHg)	163.5 ± 24.2	160.8 ± 26.8	0.512
Initial DBP (mmHg)	92.7 ± 19.2	92.2 ± 21.3	0.869
Hypertension (*n* (%))	57 (69.5)	78 (61.4)	0.232
Current smoking (*n* (%))	29 (35.4)	38 (29.9)	0.376
Atrial fibrillation (*n* (%))	21 (25.6)	25 (19.7)	0.313
Lobar hemorrhage (*n* (%))	20 (24.4)	25 (19.7)	0.419
Infratentorial hemorrhage (*n* (%))	16 (19.5)	21 (16.5)	0.582
Intraventricular hemorrhage (*n* (%))	21 (25.6)	28 (22.0)	0.553
Admission NIHSS	15.5 (9.0)	7.0 (8.0)	<0.001
Hematoma volume (mL)	19.9 (12.0)	15.8 (14.5)	0.005
WBC (×10^9^/L)	10.2 ± 2.8	9.4 ± 1.8	0.038
Serum glucose (mmol/L)	8.0 ± 1.7	7.1 ± 1.2	0.041
Serum CRP (mg/L)	25.6 (6.9)	19.3 (9.8)	<0.001
Serum TNF-*α* (pg/mL)	69.0 (20.0)	56.2 (22.0)	<0.001
Serum IL-12 (pg/mL)	79.0 (17.0)	65.3 (28.6)	<0.001

## Data Availability

The data used to support the findings of this study are available from the corresponding author upon request.

## Supplementary Materials

Supplementary Materials Figure S1. Research flow chart of this study.
