# Supplementary figures and images for: Relationship of Serum IL-12 to Inflammation, Hematoma Volume, and Prognosis in Patients With Intracerebral Hemorrhage
Source: Emerg Med Int. 2022 Oct 18;2022:8688413. doi: 10.1155/2022/8688413 (PMC9596260; doi:10.1155/2022/8688413)

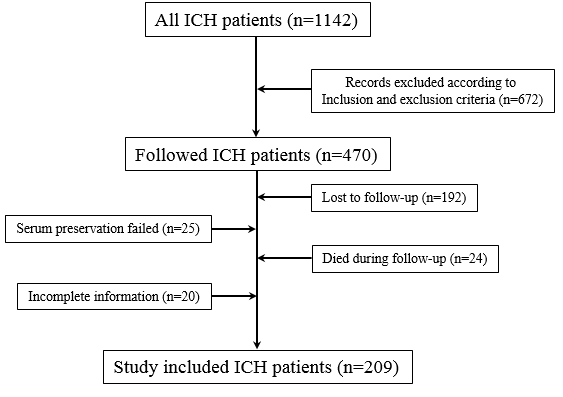


Figure S1 Research flow chart of this study.

Supplement: Supplementary Materials — Figure S1. Research flow chart of this study. [file 8688413.f1.docx]
